# Immunometabolic Pathways: Investigating Mediators of Major Depressive Disorder and Atherosclerotic Cardiovascular Disease Comorbidity

**DOI:** 10.1016/j.bpsgos.2025.100528

**Published:** 2025-05-08

**Authors:** Angela Koloi, Nabila P.R. Siregar, Rick Quax, Antonis I. Sakellarios, Femke Lamers, Arja Rydin, Kevin Dobretz, Costas Papaloukas, Dimitrios I. Fotiadis, Jos A. Bosch

**Affiliations:** a Unit of Medical Technology and Intelligent Information Systems, Department of Materials Science and Engineering, University of Ioannina, Ioannina, Greece; b Department of Biological Applications and Technology, University of Ioannina, Ioannina, Greece; c Department of Clinical Psychology, University of Amsterdam, Amsterdam, the Netherlands; d Department of Medical Psychology, Amsterdam University Medical Center, Amsterdam, the Netherlands; e Computational Science Laboratory, Institute of Informatics, University of Amsterdam, Amsterdam, the Netherlands; f Biomedical Engineering of the Department of Mechanical Engineering and Aeronautics, University of Patras, Patras, Greece; g Department of Psychiatry, Amsterdam University Medical Center, VU Amsterdam, Amsterdam, the Netherlands; h Amsterdam Public Health, Mental Health Program, Amsterdam, the Netherlands; i Cardiology, Geneva University Hospitals, Geneva, Switzerland; j Department of Medicine, University of Geneva, Geneva, Switzerland; k Biomedical Research Institute, Foundation for Research and Technology - Hellas, Ioannina, Greece

**Keywords:** Biomarkers, CVD, Machine learning, MDD, Mediators

## Abstract

**Background:**

Major depressive disorder (MDD) and cardiovascular diseases (CVDs) often co-occur whereby comorbidity results in poorer clinical outcomes, presumably due to shared immunometabolic pathways. Identifying shared biomarkers for MDD-CVD comorbidity may provide targets for prevention or treatment.

**Methods:**

Using data from the NESDA (Netherlands Study of Depression and Anxiety) (*n* = 2256, 66.3% female, mean age 41.86 ± 13.08 years at baseline), validated with the UK Biobank (UKB) data (*n* = 35,668, 56.14% female, mean age 63.95 ± 7.74 years), this study aimed to identify 1) biomarkers, closely associated with current MDD, and 2) longitudinal pathways linking MDD and atherosclerotic CVD. Plasma metabolites (nuclear magnetic resonance) and inflammatory markers were used as exposures within a machine learning framework. Influential biomarkers were integrated into a temporal network analysis linking MDD to subsequent CVDs, exploring longitudinal pathways through causal discovery, validated by sensitivity analysis and centrality assessment. External validation included mediation and regression analysis adjusting for covariates.

**Results:**

Network analysis identified stable direct paths from MDD to CVDs via tumor necrosis factor α (TNF-α), tyrosine, and fatty acids and indirect paths via acetate, high-density lipoprotein (HDL) diameter, interleukin 6, AGP, high-sensitivity C-reactive protein, and low-density lipoprotein triglycerides. Among these, acetate, tyrosine, AGP (α_1_-acid glycoprotein), and HDL diameter potentially mediated the MDD-CVD connection, given that these were identified as key nodes within the network. UKB validation confirmed HDL diameter (β = 0.004) and AGP (β = 0.003) as significant depression-CVD mediators (both *p* < .001), after adjusting for age, sex, deprivation index, alcohol consumption, smoking status, physical activity, and body mass index.

**Conclusions:**

These analyses identified biomarkers shared in MDD and CVDs and may drive comorbid pathology risk.

## Plain Language Summary

Depression and heart disease often occur together, leading to worse health outcomes, but the biological reasons for this link are not fully understood. This study investigated whether shared immune and metabolic pathways might connect these conditions. Using data from 2 large health studies, we identified key blood markers involved in both depression and heart disease. We found that inflammation-related proteins (such as) (e.g., *AGP* [α1-acid glycoprotein]) and cholesterol particles (high-density lipoprotein [HDL] diameter) may play a role in this connection. These findings could help develop better treatments to reduce the risk of heart disease in people with depression.

Major depressive disorder (MDD) is a complex psychiatric disorder, exhibiting a chronic or recurrent course in a significant proportion of afflicted individuals. As the most prevalent mood disorder worldwide, it is the leading cause of disability, affecting an estimated 322 million people worldwide (1). MDD significantly reduces individual productivity and imposes substantial economic costs (2). Its incidence continues to rise, with no indications of leveling off, underscoring that depression is one of the most formidable public health challenges (3).

MDD often co-occurs with cardiovascular diseases (CVDs) (4,5), resulting in worse clinical outcomes marked by higher disease severity and increased mortality risk (6). Each condition significantly increases the risk of developing the other, highlighting a bidirectional relationship between CVDs and depression (5,6). The fact that both conditions involve immunoinflammatory and metabolic dysregulations has led to the proposition of metabolic and immune system changes as a comorbidity pathway (7). For example, large-scale metabolomic meta-analysis has revealed that depression is associated with a distinctive immunometabolic signature involving elevated levels of CVD risk factors such as AGP (α_1_-acid glycoprotein), triglycerides, and very-low-density lipoproteins and lower high-density lipoproteins (HDLs) (8). Similarly, systematic review (9) and meta-analysis (10,11) on inflammation’s role in CVDs have reported findings that are consistent with those observed in depression, suggesting a potential connecting substrate or shared mechanism between depression and CVDs. Initially based on experimental evidence, the cytokine theory of depression (12) proposes a causal role of inflammation through inducing sickness behavior (13), characterized by symptoms such as weight loss, fatigue, sleepiness, and failure to concentrate. Subsequent meta-analyses (14,15) and gene expression (16,17) studies of observational data have supported that depression is consistently linked with elevated levels of inflammatory markers.

Research into MDD-CVD comorbidity highlights 2 critical needs: although much research studied each condition and their associated biological pathways in separation, while making extrapolations to the other, a truly integrated approach, studying these and their shared inflammatory and metabolic pathways together, is virtually lacking. Second, there is a paucity of longitudinal analyses that explore how these chronic conditions codevelop over time. This study addressed these needs guided by 2 hypotheses. The first is that depressive episodes (compared with quiescent periods) are characterized by specific cardiopathological metabolites and inflammatory markers. We used machine learning (ML) to identify most characteristic markers and assess their significance for cardiopathology.

The second hypothesis proposes that the cardiometabolic factors elevated in depression are predictive of subsequent CVDs. However, such analyses are sparse. To test these hypotheses, a temporal network analysis framework, informed by ML insights, was constructed to uncover both direct and indirect pathways between MDD and subsequent CVD comorbidity. In the absence of experimental data, the role of such pathways may be best studied using unbiased data-driven analyses in longitudinal cohorts.

To identify comorbid pathways, a causal graph (CG) was constructed and represented as a directed acyclic graph. The structure of the CG was identified through causal discovery, which infers relationships directly from data when the underlying causal structure is unknown (18, 19, 20). For this study, we used the tiered Peter-Clark (tPC) algorithm (21), selected for its ability to account for the temporal order of variables.

## Methods and Materials

### Netherlands Study of Depression and Anxiety

The NESDA (Netherlands Study of Depression and Anxiety) is a longitudinal cohort that consists of data on depression and anxiety disorders and demographic, psychological, physiological, and genetic information (22). Participants ages 18 to 65 years were recruited from various settings in the Netherlands between 2004 and 2007, including community, primary care, and specialized mental health care. The sample consists of adults with a current or lifetime diagnosis of anxiety or depressive disorder (as determined with the DSM-IV–based Composite International Diagnostic Interview [CIDI], lifetime version 2.1) (23) and healthy control participants. The current analysis focuses on 2 specific waves: the initial NESDA wave, which serves as the baseline with 2981 participants, and the fifth NESDA wave, designated as the follow-up, with 2256 participants.

### Exposures

#### Metabolomic Biomarker Measurement

Participants provided fasting blood samples during the baseline interview. Assessments were based on a proton nuclear magnetic resonance (NMR) platform (Nightingale Health Plc) that quantified concentrations of several lipid-related metabolites and their ratios (24). Among the measures provided by the platform, we selected concentrations of 51 metabolites as in a previous study (25). Additional lipoprotein submeasures were excluded due to redundancy. Data were preprocessed per the manufacturer’s protocol (8), with a natural log transformation applied after adding 1 to each value. Data points exceeding 5 SDs from the mean were treated as missing.

#### Inflammatory Marker Measurement

At baseline NESDA assessment, inflammatory markers—high-sensitivity C-reactive protein (hs-CRP), interleukin 6 (IL-6), and tumor necrosis factor α (TNF-α)—were measured due to their established links to depressive symptoms and MDD (14, 15, 16,26). hs-CRP was assayed using an in-house enzyme-linked immunosorbent assay (ELISA) with Dako reagents, IL-6 with PeliKine Compact ELISA (Sanquin), and TNF-α using Quantikine HS Immunoassay (R&D Systems) (see the Supplement for full assay details). All markers required log transformation for analysis as they violated normality assumptions. Complete details of all biomarkers—categorized by analytical method (inflammatory assays vs. metabolic profiling)—are presented in Table S1.

### Outcomes

#### Major Depressive Disorder

Diagnosis of current MDD was based on the CIDI, a standardized diagnostic tool used globally to classify psychiatric disorders according to DSM-IV criteria (23). In this study, current (e.g., in the past 6 months) MDD diagnoses at baseline served as the outcome. Longitudinal diagnostic patterns showed 1115 participants with current MDD at NESDA baseline, decreasing to 358 cases at the 6-year follow-up. The observed MDD prevalence reduction reflects the disorder’s natural course, with most baseline cases remitting by follow-up. Attrition played only a minor role.

#### Cardiovascular Diseases

Self-reported CVDs was based on the reported presence of heart diseases, stroke, and surgical interventions. Diseases included coronary heart disease, angina pectoris, heart failure, cardiac arrhythmia, and other heart diseases (see the Supplement). Medication confirmation followed strict Anatomical Therapeutic Chemical classification (B01, C01D, C02-C04, C07-C09, N02BA01/15 <100 mg). Only participants with both self-reported CVDs and corroborating medications were classified as cases. In this study, CVDs identified at the 6-year follow-up served as the outcome measure. Of the participants evaluated at the NESDA follow-up, 210 had CVDs.

### UK Biobank

The UK Biobank (UKB) is a population-based cohort study comprising approximately 500,000 UK residents ages 40 to 69 years who were recruited between 2006 and 2010 (27). UKB provides a robust dataset for external validation of NESDA findings, featuring key variables such as metabolic biomarkers quantified using the same NMR platform as NESDA. This consistency extends to the assessment of depression, with both studies using the CIDI for MDD diagnoses (current for NESDA; lifetime for UKB).

### Exposures

#### Metabolomic Biomarkers

The NMR dataset included detailed metabolic biomarkers, quantified from 118,461 baseline plasma samples, processed by Nightingale Health Plc. This large-scale dataset represents a substantial improvement over previous metabolic profiling studies, with a sample size more than 10 times larger than many earlier efforts.

### Outcomes

This study included up to 157,286 UKB participants with available depression and anxiety data (28). Current depression status was determined through a composite approach combining 1) CIDI-derived lifetime MDD criteria from the Mental Health Questionnaire and 2) current depressive symptoms assessed by the Patient Health Questionnaire-9 (PHQ-9) (29). For PHQ-9 classification, participants met all 3 criteria: 1) total score ≥10 (range: 0–27), 2) ≥5 symptoms occurring more than half the days, and 3) either anhedonia or depressed mood with scores ≥2 (on 0–3 scales); probable bipolar cases were excluded. The PHQ-9 was particularly valuable for identifying current cases because the CIDI items in UKB lack temporal resolution for current episodes.

Depression prevalence differed by assessment method: 29.4% of participants had CIDI-derived lifetime diagnosis alone, 1.1% met PHQ-9 criteria only (see Table S2 for symptom frequencies and missingness), and 2.3% (*n* = 2900) met both criteria and were classified as currently depressed.

UKB used provider-diagnosed atherosclerotic CVDs defined by ICD-10 codes for acute events (I21-I22, I63), chronic manifestations (I25.2, I70.2), and procedures (Z95.1, Z95.5), excluding nonatherosclerotic conditions (Table S3). While methodological differences existed between cohorts, both prioritized hard atherosclerotic end points to enhance comparability. In UKB, 6.68% of participants met CVDs criteria, which was encoded as a binary variable (1 = present, 0 = absent) for all analyses.

### Covariates

In NESDA, we intentionally omitted lifestyle covariates to focus specifically on immunometabolic pathway testing. For UKB analyses, we adopted a more comprehensive adjustment strategy that included 1) sociodemographic factors [age, sex, Townsend Deprivation Index (TDI) derived from postcode-linked census data (30)], 2) behavioral factors (self-reported smoking status, alcohol consumption, and weekly moderate physical activity), and 3) anthropometric measures (body mass index [BMI]). This covariate-rich approach in UKB served to test the robustness of observed associations (see the Supplement for full measurement details).

### Preprocessing Analysis

**Figure 1:**
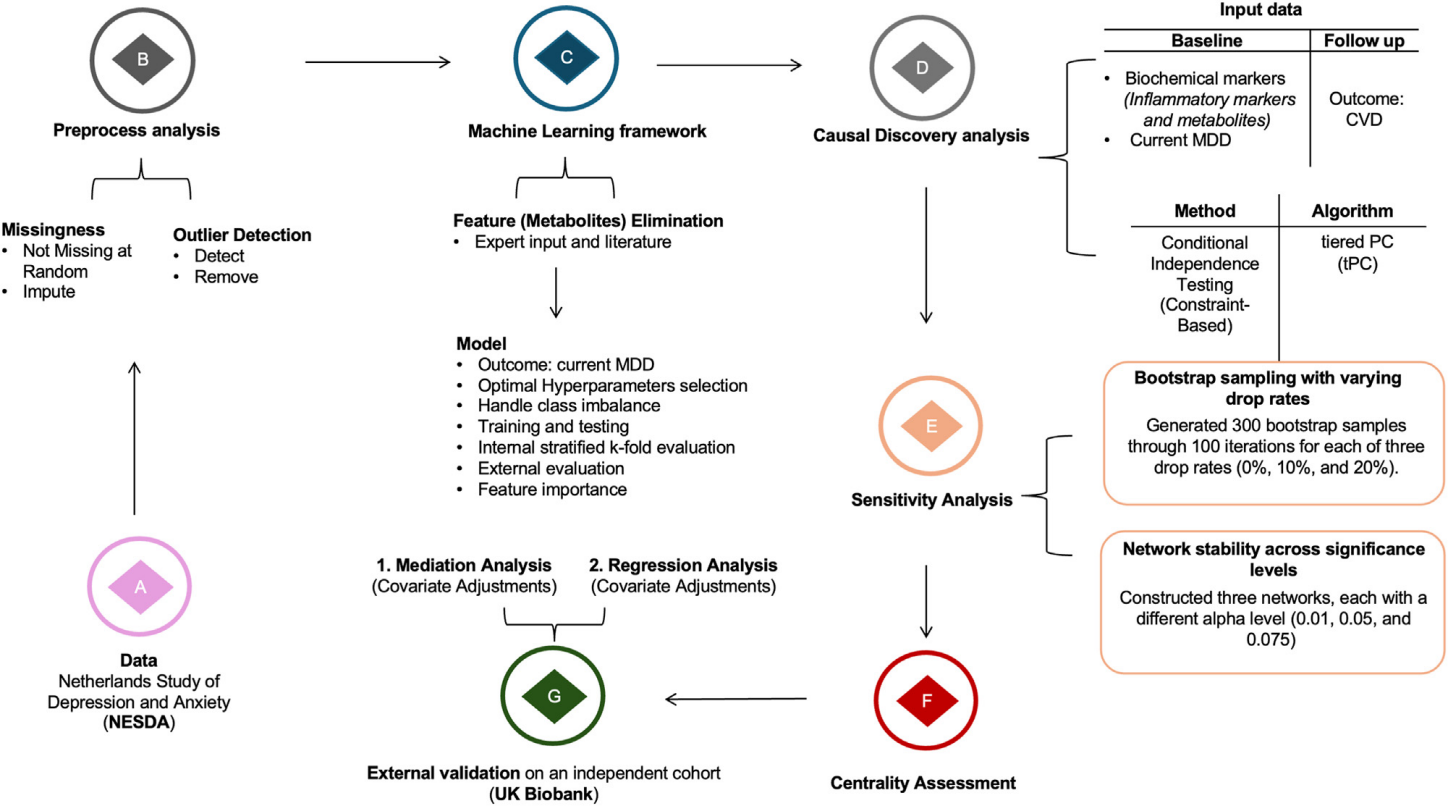
Analysis pipeline began with **(A)** data from the NESDA, which provided the foundation for the research question, focusing on biochemical markers and major depressive disorder (MDD) at baseline and cardiovascular disease (CVD) outcome at follow-up. Data preprocessing **(B)** included handling missingness (assumed to be not missing at random) through imputation, along with outlier detection, validation, and removal. The prediction model framework **(C)** involved feature selection guided by expert input and literature, with MDD prediction using XGBoost, accounting for optimal hyperparameter selection, handling class imbalance, training, testing, evaluation, and assessing feature importance stability via bootstrap sampling. Causal discovery analysis **(D)** used the tiered PC algorithm for constraint-based conditional independence testing to explore relationships between biomarkers and outcomes. Sensitivity analysis **(E)** evaluated the robustness of findings using 300 bootstrap samples with varying drop rates (0%, 10%, 20%) and network stability across 3 significance levels (0.01, 0.05, 0.075) and **(F)** centrality assessment calculated betweenness centrality. The final step **(G)** involved external validation on an independent cohort (UK Biobank) to verify the robustness and generalizability of the identified biomarkers through mediation and regression analyses.

An overview of the analysis steps is depicted in Figure 1. Based on the results of Little’s missing completely at random test (31), the missingness was not completely at random; hence, we could not drop these values (Table 1). Due to its ability in capturing complex relationships, we performed multivariate imputation using the iterative imputer from scikit-learn in Python version 3.11.4. We applied the isolation forest algorithm (32) to detect outliers (Supplemental Methods). After identifying these outliers, we used box plots to visualize their distribution. Outliers (*n* = 118) were then removed from the dataset to improve data quality and ensure that the analysis was centered on representative, nonskewed samples. To maximize statistical power, all NESDA participants were included in the analyses. Folow-up CVD measures were residualized for CVD baseline status, age, and sex effects using logistic regression, thereby isolating the component of cardiovascular outcomes independent of these factors.

**Table 1:** Descriptive Statistics in NESDA

Characteristics	Baseline, *n* = 2981		Follow-Up, *n* = 2256	
	Mean (SD), %, or *n*	Missing	Mean (SD), %, or *n*	Missing
Sociodemographics				
Age, Years	41.86 (13.08)	0%	47.80 (13.11)	0%
Sex, Female	66.38%	0%	66.31%	0%
Cardiovascular Indicators				
Heart Diseases				
Heart condition/heart infarct^a^	121	0%	108	0.2%
Stroke				
Did you ever have a stroke (CVA)? Use of medication for disease	31	0%	32	0.2%
Heart Operation				
Did you have an operation on your heart or coronary artery?				
Heart valve	6	0%	9	0.2%
Bypass	17		17	
Balloon treatment	40		27	
Pacemaker	2		4	
Other	16		13	
Psychological Indicator				
Current Major Depressive Disorder	1115	0%	358	0%

CVA, cerebrovascular accident; NESDA, Netherlands Study of Depression and Anxiety.

a Coronary heart disease, angina pectoris, heart failure, cardiac arrhythmia, other heart disease, and all medication adjusted.

### ML Framework

We started by a feature elimination process including metabolites from the NMR platform and 3 inflammatory markers at baseline as in previous studies (16,25). A multidisciplinary team of 10 researchers and clinicians, including psychiatrists and cardiologists, was engaged throughout the study. These experts collaborated to refine the primary research questions and identify characteristics for inclusion. While the selection of inflammatory markers was straightforward due to the limited availability of only 3 markers in NESDA, the team’s expertise was instrumental in narrowing down the list of relevant metabolites for analysis. The markers were narrowed down to a total of 54 biomarkers based on the overlap between expert input and relevant literature (8,16). To further refine the number of metabolites, we applied an eXtreme Gradient Boosting (XGBoost) classifier, an ML algorithm, to pinpoint metabolites strongly associated with current MDD at baseline, under the hypothesis that these markers are elevated during a depressive episode (15). For details on model imbalance, hyperparameter optimization, feature importance assessment, and stability analysis of feature importance, see Supplemental Methods.

### CG Analysis

We used a temporal network analysis model to investigate direct/indirect paths between current MDD, refined markers at baseline, and CVDs at follow-up. We used a constraint-based causal discovery (33) algorithm (Supplement) called the tiered PC from the tPC package (21,34), due to its suitability for temporal data. This algorithm, a modified version of the PC algorithm (35), accommodates node assignments across different tiers (levels representing distinct time points) and was selected for its ability to handle partial node ordering, making it well suited for longitudinal data. The tPC algorithm is well suited for generating empirically plausible directed acyclic graphs, effectively capturing both direct and indirect relationships. For independence testing in the tPC algorithm, we used the likelihood ratio test (36) for conditional independence in mixed data from the micd package (34) with an alpha threshold of 0.05, suitable for datasets with both continuous and categorical variables, assuming a conditional Gaussian distribution.

#### Sensitivity Analysis

To assess the robustness and stability of our findings, we conducted a sensitivity analysis using bootstrap sampling combined with varying drop rates. Specifically, we generated a total of 300 bootstrap samples by performing 100 bootstrap iterations for each of the 3 sample drop rates: 0%, 10%, and 20%. Each bootstrap sample was generated by randomly selecting observations with replacement from the preprocessed dataset, allowing us to capture sampling variability and its impact on the model. For each iteration, we applied the tPC algorithm.

To further assess the validity of our findings, we conducted an additional sensitivity analysis by constructing 3 separate networks, each using a different significance level (alpha) for edge inclusion in the network structure: 0.01, 0.05, and 0.075. The visualizations display 3 interactive networks, each representing different alpha levels in the sensitivity analysis. Nodes are color coded by group, differentiating between the MDD and CVD outcomes, metabolites, and inflammatory markers.

#### Centrality Assessment

To assess the importance of individual nodes/biomarkers within the network, we used betweenness centrality, a commonly used metric in network analysis (37). Betweenness calculates how often a node appears on the shortest paths between other nodes, where shortest paths refer to the minimal number of edges connecting 2 nodes. Nodes exhibiting high betweenness centrality lie on the paths linking many node pairs, thus playing a crucial role in mediating or managing information flow throughout the network (38).

### External Validation on UKB

#### Mediation Analysis

We conducted external validation of the stable and central biomarkers using cross-sectional UKB, performing formal mediation analysis (39) that adjusted for sociodemographic factors (age, sex, TDI), behavioral covariates (smoking status, alcohol consumption, physical activity), and BMI to quantify each biomarker’s mediating effect size in the depression-CVD association. The analysis used a 2-model approach: first examining the depression-biomarker relationship using ordinary least squares regression and then testing the combined effect of depression and biomarker on CVDs using logistic regression.

The average causal mediation effect (ACME) quantified the magnitude of MDD’s indirect influence on CVD risk through each biomarker pathway. We considered mediation to be statistically significant when ACME estimates demonstrated *p* values < .05, rejecting the null hypothesis of no mediation effect. Sensitivity analysis involved a bootstrapping approach with 1000 repetitions to improve estimate reliability and generate confidence intervals for the indirect effects, further strengthening the robustness of the analysis (40).

#### Covariate Adjustment

Two logistic regressions were performed, with CVDs and current depression as the dependent variables and biomarkers as the independent variables in the first model. Both models were adjusted for covariates, including age, sex, TDI, alcohol consumption, smoking status, physical activity, and BMI.

## Results

### Preprocessing

Descriptive statistics and the extent of missing data for the variables of NESDA are presented in Table 1. Outliers, predominantly extreme values in inflammatory markers suggestive of acute inflammation, were visualized using box plots (Figure S1) and subsequently excluded to enhance data quality, resulting in a reduction of the initial sample size from 2256 to 2138. Overlap of expert input and literature narrowed the biomarkers down to 54 (Table S1). The performance metrics of the XGBoost model for classifying current MDD are summarized in Table S4. For detailed model performance metrics, see Supplemental Results. With regard to our first aim, we concluded on 24 biomarkers associated with current MDD. Descriptive statistics and missingness of the refined biomarkers are presented in Table S5. The biomarker distributions in their raw, pre–log-transformation state are presented in Figure S2. The sorted feature importance of the biomarkers with error bars representing the SD is presented in Figure S3A, while the receiver operating characteristic curve of the XGBoost model (area under the curve = 0.84) is presented in Figure S3B. Descriptive statistics and the extent of missing data for UKB are presented in Table 2. After applying the data cleaning steps to the UKB data as those used for NESDA, the NMR metabolomic data (*n* = 118,461) were combined with UKB participants who had completed the relevant questionnaires (*n* = 157,286), resulting in a final sample of 35,668 patients.

**Table 2:** Descriptive Statistics in the UK Biobank

Characteristics	UK Biobank, *n* = 157,286	
	Mean (SD) or %	Missing
Sociodemographics		
Age, Years	64.05 (7.73)	0%
Sex, Female	56.2%	0%
Townsend Deprivation Index		
Average	31.24%	0.12%
Most	12.28%	
Least	56.48%	
Lifestyle and Health		
Smoking Status		
Prefer not to answer	0.2%	0.04%
Never	57.45%	
Former	35.14%	
Current	7.21%	
Alcohol Consumption		
Never	8.90%	0.12%
Monthly or less	13.25%	
2–4 times a month	18.34%	
2–3 times a week	29.75%	
4 or more times a week	29.96%	
Moderate Physical Activity	87.69%	2.8%
Body Mass Index	26.79 (4.55)	1.43%
Atherosclerotic CVD	6.68%	0%
Current Depression Classification	2.3%	19.96%
CIDI lifetime diagnosis	29.4%	
PHQ-9 depression classification^a^	1.1%	

CIDI, Composite International Diagnostic Interview; CVD, cardiovascular disease; PHQ-9, Patient Health Questionnaire-9.

a PHQ-9 depression classification met all 3 criteria: 1) total score ≥10 (of 27 possible), 2) ≥5 symptoms occurring more than half the days, and 3) either anhedonia or depressed mood ≥2 (on 0–3 scales).

### CG Analysis, Stability, and Centrality

**Figure 2:**
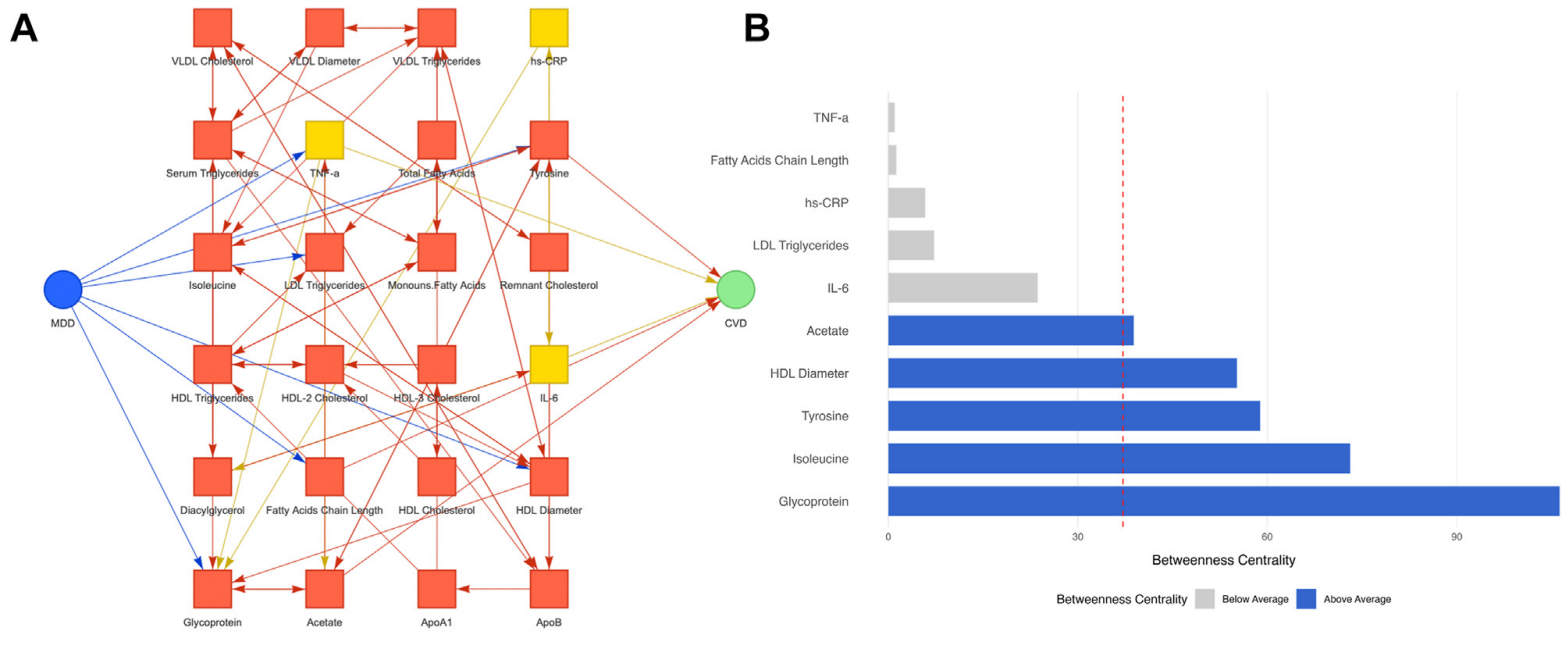
**(A)** Significant pathways linking baseline major depressive disorder (MDD) to incident cardiovascular diseases (CVDs) through immunometabolic mediators. Blue node, MDD; green, CVD; yellow, inflammatory markers; orange, metabolic markers. **(B)** The bars represent the betweenness centrality scores of the nodes found as intermediates between current MDD and CVDs in the longitudinal network. Nodes with a betweenness centrality above the mean are highlighted in blue, while those below the mean are in gray. The dashed red line indicates the mean betweenness centrality. HDL, high-density lipoprotein; hs-CRP, high-sensitivity C-reactive protein; IL-6, interleukin 6; LDL, low-density lipoprotein; TNF-α, tumor necrosis factor α; VLDL, very-low-density lipoprotein.

The network included baseline measures of current MDD and refined biomarkers (comprising 24 variables), while the follow-up outcomes focused on CVDs, based on a sample of 2138 participants after outlier removal. At baseline, 1115 participants met diagnostic criteria for current MDD. Of these, 210 developed incident CVDs during follow-up. The significant biological pathways connecting baseline MDD to subsequent CVDs are presented in Figure 2A.

Stability analysis highlighted strong connections in pathways from current MDD (cause) to key biomarkers (effect). The stability assessment results from the NESDA data are summarized in Table 3, which displays the percentage frequency of appearance in 300 bootstrapped graphs, and 3 significance levels (alpha) tested to evaluate consistency across conditions. Interactive network visualizations are available at 3 statistical thresholds: link1 (α = 0.01, most conservative), link2 (α = 0.05, primary analysis), and link3 (α = 0.075, most inclusive). These reveal robust MDD-to-CVD pathways through both direct mediators (TNF-α, IL-6, tyrosine, and fatty acids [FAs]) and indirect pathways (acetate, HDL diameter, AGP, hs-CRP, isoleucine, and low-density lipoprotein [LDL] triglycerides). The 10 identified biomarkers showed high consistency, appearing in 80% to 100% of bootstrapped graphs (median, 94%). Complete bootstrap results are provided in Supplemental Results.

**Table 3:** Edge Stability Assessment

Cause	Effect	Stability Analysis			
		Significance Level, α			% Frequency in 300 Graphs
		α = 0.01	α = 0.05	α = 0.075	
MDD	Acetate	*✔*	*✔*	*✔*	88%
	Tyrosine	*✔*	*✔*	*✔*	98.3%
	HDL diameter	*✔*	*✔*	*✔*	95%
	TNF-α	*✔*	*✔*	*✔*	93%
	IL-6	–	–	*✔*	86%
	AGP	*✔*	*✔*	*✔*	100%
	hs-CRP	*✔*	*✔*	*✔*	80%
	Isoleucine	*✔*	*✔*	*✔*	94.6%
	FA chain length	*✔*	*✔*	*✔*	99.6%
	LDL triglycerides	–	*✔*	*✔*	87%
Acetate	CVD	*✔*	*✔*	*✔*	99.7%
Tyrosine		*✔*	*✔*	*✔*	96.7%
HDL Diameter		–	–	–	96%
TNF-α		*✔*	*✔*	*✔*	96.3%
IL-6		*✔*	*✔*	*✔*	99.3%
AGP		*✔*	*✔*	*✔*	93%
hs-CRP		*✔*	*✔*	*✔*	97.3%
Isoleucine		*✔*	*✔*	*✔*	98.6%
FA Chain Length		*✔*	*✔*	*✔*	97.3%
LDL Triglycerides		–	–	–	82.3%

The table presents direct/indirect pathways between current MDD (first cause) and biomarkers (effect) and biomarkers (second cause) and the outcome CVD (effect) evaluated for stability across varying significance levels (α) of 0.01, 0.05, and 0.075. The last column indicates the frequency with which each pathway link appeared across 300 bootstrapped samples, providing an additional measure of edge stability.

CVD, cardiovascular disease; FA, fatty acid; HDL, high-density lipoprotein; hs-CRP, high-sensitivity C-reactive protein; LDL, low-density lipoprotein; MDD, major depressive disorder; TNF-α, tumor necrosis factor α.

Centrality analysis was used to assess the role of biomarkers within the network. For the 10 stable biomarkers, we identified tyrosine, acetate, HDL diameter, AGP, and isoleucine as highly central (potential mediators) based on their high betweenness centrality scores (Figure 2B).

### External Validation

**Figure 3:**
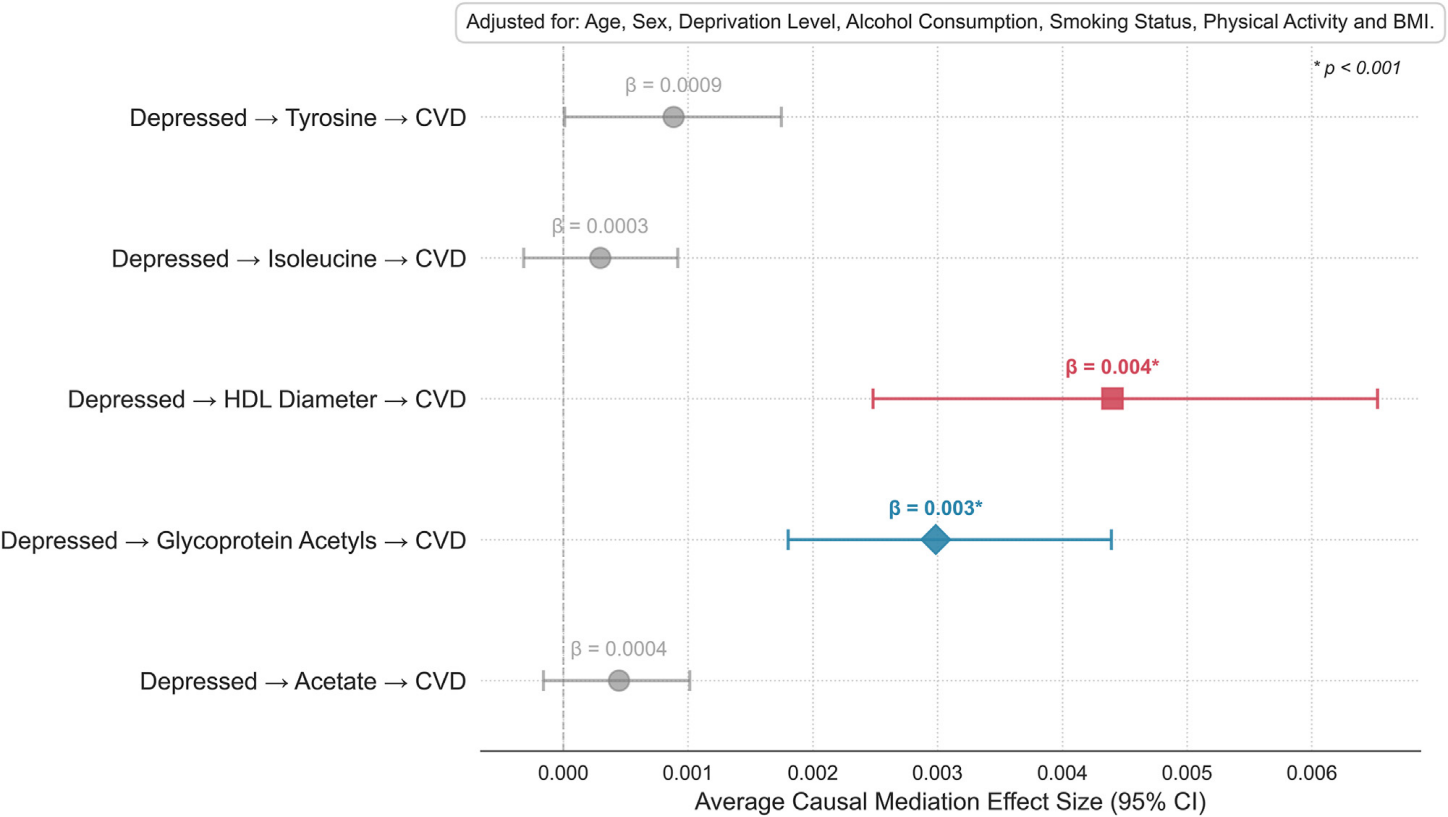
Forest plot of mediation analysis results in the UK Biobank, examining potential mediators between depression (exposure) and atherosclerotic cardiovascular disease (CVD) outcomes. Mediators include acetate, glycoprotein acetyls, high-density lipoprotein (HDL) diameter, isoleucine, and tyrosine. Significant pathways (*p* < .001) are highlighted with colored markers, while nonsignificant pathways are displayed in gray. Adjusted covariates include age, sex, deprivation level, alcohol consumption, smoking status, physical activity, and body mass index (BMI). The average causal mediation effect sizes with 95% CIs are shown for each pathway.

To externally validate the identified intermediates, we performed mediation analyses using UKB, adjusting for covariates. As shown in Figure 3, HDL particle diameter (β = 0.004, *p* < .001) and AGP (β = 0.003, *p* < .001) demonstrated significant mediation effects in the depression–atherosclerotic CVD pathway. In contrast, acetate (*p* = .08), tyrosine (*p* = .816), and isoleucine (*p* = .648) showed no significant mediation.

**Figure 4:**
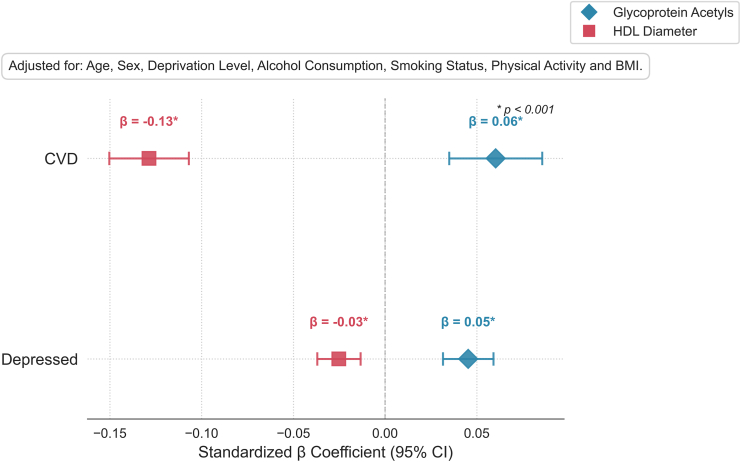
Forest plot of regression analysis results in the UK Biobank, showing associations between glycoprotein acetyls and high-density lipoprotein (HDL) diameter with 2 independent variables: current depression and atherosclerotic cardiovascular disease (CVD). The standardized β coefficients are plotted with 95% CIs. Covariates adjusted in the model include age, sex, deprivation level, alcohol consumption, smoking status, physical activity, and body mass index (BMI). Significant results (*p* < .001) are marked with an asterisk (∗).

An additional external validation analysis (regression analysis), using data from the UKB again, assessed the impact of stable, central, and significant mediator biomarkers through covariate adjustments. As shown in Figure 4, results for CVDs indicated that HDL particle diameter demonstrated a negative association with a beta coefficient of −0.13 (*p* < .001), and AGP showed a positive association with a beta of 0.06 (*p* < .001). In the case of current MDD, the associations were as follows: HDL particle diameter exhibited a beta of −0.03 (*p* < .001) and AGP had a beta of 0.05 (*p* < .001).

## Discussion

Using a multimodal data analysis framework, this study aimed to identify biomarkers associated with current MDD and to elucidate shared immunometabolic pathways linking MDD with CVDs. The key innovation lies in the use of ML, longitudinal network, and mediation analysis to connect MDD and subsequent CVDs, which are typically studied separately. By externally validating findings, we further verified the reliability of our results.

With regard to the first hypothesis, our study used expert consultation, literature review, and ML techniques to identify key biomarkers that are significantly influential in MDD. The model classified individuals based on their current MDD status, distinguishing those with MDD from those without. Our findings revealed that 21 metabolites—apolipoproteins, cholesterol, FAs, glycerides, lipoproteins, amino acids, and ketone bodies—and 3 inflammatory markers exhibit a high influential value for MDD. Notably, this biological signature is partially shared with CVDs that commonly co-occur with depression (4,5).

Studies have shown that individuals with long-term depression exhibit lower HDL cholesterol (HDL-C) levels and higher atherogenic indices than healthy control participants (41). Elevated triglycerides reflect impaired lipid metabolism, with chronic stress and hypothalamic-pituitary-adrenal axis dysregulation in MDD thought to contribute to these lipid abnormalities (42). Both apolipoprotein B and triglycerides are linked to oxidative stress, which accelerates cellular damage and inflammation, likewise connecting depression (8,43). Interestingly, studies have observed lower isoleucine, a branched-chain amino acid, levels in individuals with depression, suggesting a potential role in mood disorders (44). In addition to metabolites, our analysis found TNF-α, hs-CRP, and IL-6 to be significantly influential in MDD. Elevated levels of inflammatory markers have been linked to depressive symptoms (16). Notably, a review of experimental models and clinical trials provided evidence that the use of TNF-α antagonists could offer a novel therapeutic approach for alleviating cognitive dysfunction and depressive symptoms in patients with MDD (45).

Regarding our second aim, the analysis revealed direct pathways longitudinally linking MDD to CVDs via biomarkers such as TNF-α, IL-6, tyrosine, and FAs, as well as indirect pathways involving acetate, HDL diameter, AGP, hs-CRP, isoleucine, and LDL in triglycerides. Understanding the distinction between direct and indirect pathways is crucial, because it demonstrates how MDD can influence CVDs. Direct pathways, which could be described as single-mediator pathways, represent straightforward relationships (MDD→TNF-α→CVD). In contrast, indirect pathways, which could be termed multimediator pathways, involve complex interactions with multiple intermediaries (MDD→AGP→acetate→CVD). Betweenness assessment within the network identified tyrosine, acetate, HDL diameter, AGP, and isoleucine as central. Cross-sectional validation with data from the UKB confirmed the significance of AGP and HDL diameter as potential mediators in the MDD-CVD relationship, with their importance reinforced after adjusting for covariates.

AGP was found to be a potential mediator in MDD-CVD, underscoring the role of chronic (low-grade) inflammation in their comorbidity. Clinical (6) and narrative (46) reviews pinpoint the significance of inflammatory processes in the depression-CVD relationship. AGP is an acute-phase protein that increases in response to inflammation, playing a role in immune modulation and compound transport in the bloodstream (47). Elevated AGP levels in patients with depression suggest its potential as a biomarker (8). Research indicates that AGP is more stable and predictive of future CVD risks, such as hypertension and metabolic syndrome in young individuals, than hs-CRP (48), while a review study supports that biomarkers such as GlycA and GlycB could be superior for early detection and prediction of cardiometabolic diseases and inflammation (49).

HDL diameter emerged as a potential mediator in the MDD-CVD relationship, underscoring the role of lipid dysregulation in their comorbidity. HDL-C has traditionally been used as a lipid marker in assessing CVD risk, but its causal role in atherosclerotic CVD remains debated. While some studies have indicated that high HDL-C correlates with lower CVDs and mortality (50), recent genetic and cohort studies have suggested that elevated HDL-C levels do not consistently reduce risk and may even increase it in certain contexts (51). Recent studies have suggested that HDL particle number and diameter may offer more precise predictions of cardiovascular risk than traditional HDL-C measurements, potentially improving cardiovascular risk assessments (52,53). A fair body of research on HDL-C and depression provides insights into the complex relationship between lipid metabolism and mental health (8,54). However, this relationship is also not straightforward, given that some studies have found a positive correlation between HDL levels and depression (55), whereas others have shown an inverse relationship (8,41).

Previous studies have linked MDD and CVDs through shared pathways, with inflammation, triglycerides, and genetic factors suggesting common environmental and immunometabolic influences (54,56). This study extends previous work by using a longitudinal design to identify potential mediators between MDD and CVDs, using a large, well-characterized dataset from the NESDA, validated with an independent cohort, ensuring robust findings.

Despite its strengths, this study has several limitations. First, the observational design limits our ability to make definitive causal claims; while the graphical models reveal potential direct pathways, they cannot establish causation with certainty (57). Moreover, CVD diagnoses in NESDA were based on self-report.

Depression’s heterogeneous nature suggests that metabolic and inflammatory alterations vary depending on the specific symptom profile, with some depressive symptoms more strongly linked to metabolic changes than others (58). Future work should address depressive symptoms as the unit of analysis, rather than an MDD diagnosis, which allowed us to capture heterogeneous patterns of depression. Additionally, while we adjusted for age and sex, it will be interesting to model lifestyle factors as upstream covariates.

### Conclusions

Our findings further support and expand the current literature on immunometabolic depression (59). These metabolic disruptions may, in turn, contribute to the development of CVDs and vice versa. Our study emphasizes the significance of AGP and HDL diameter in mediating the relationship between MDD and CVDs, highlighting the shared metabolic pathways. These findings are consistent with previous research linking AGP (8,48,49) and lipid dysregulation (8,41,50,51,54,55) to both depression and CVDs. While past research has shown varying links between HDL-C, CVD risk, and depression, our study emphasizes that the significance of HDL particle diameter may serve as a more reliable biomarker. These findings highlight the potential for targeted strategies to manage this comorbidity, paving the way for preventive measures or therapies for depression and its associated CVD comorbidity.

## Supplementary Material

Supplemental Methods, Results, Figures S1–S3, and Tables S1–S6

Key Resources Table
