# Supplementary material for: Immunometabolic Pathways: Investigating Mediators of Major Depressive Disorder and Atherosclerotic Cardiovascular Disease Comorbidity
Source: Biol Psychiatry Glob Open Sci. 2025 May 8;5(5):100528. doi: 10.1016/j.bpsgos.2025.100528 (PMC12209949; doi:10.1016/j.bpsgos.2025.100528)
Supplement: Supplemental Methods, Results, Figures S1–S3, and Tables S1–S6 [file mmc1.pdf]

SUPPLEMENTARY INFORMATION

Immuno-Metabolic Pathways: Investigating Mediators of Major Depressive Disorder and Atherosclerotic Cardiovascular Disease Comorbidity

Koloi *et al.*

This document contributes as Supplement information regarding the work "Immuno-Metabolic Pathways: Investigating Mediators of Major Depressive Disorder and Atherosclerotic Cardiovascular Disease Comorbidity". This work is divided into four sections: Supplement Materials, Supplement Methods, Supplement Results, Supplement Figures and Tables.

Supplement Materials

NESDA

The NESDA is a longitudinal cohort study that consist of data on depression and anxiety disorders, demographic, psychological, physiological, and genetic information (1). Exclusion criteria were: 1) a primary clinical diagnosis of psychotic disorder, obsessive compulsive disorder, bipolar disorder or severe addiction disorder and 2) not being fluent in the Dutch language. The NESDA study follows participants across multiple data collection waves.

Exposures

Inflammatory markers measurement

Blood samples were collected in the morning, on average 5 days after participants completed the baseline Inventory of Depressive Symptomatology (IDS) and Beck Anxiety Inventory (BAI). Samples were stored at -70°C until analysis. CRP was measured in duplicate at VU University Medical Center using an in-house ELISA based on Dako (Glostrup, Denmark) purified protein and polyclonal anti-CRP antibodies (intra-assay CV: 5%, inter-assay CV: 10%; detection limit: 0.1 mg/l). IL-6 levels were determined in duplicate using PeliKine Compact high-sensitivity ELISA (Sanquin, Amsterdam, Netherlands; intra-assay CV: 8%, inter-assay CV: 12%; detection limit: 0.35 pg/ml). TNF-α was assayed in duplicate at Good Biomarker Science (Leiden, Netherlands) using Quantikine HS Human TNF-α Immunoassay (R&D Systems, Minneapolis, MN, USA; intra-assay CV: 10%, inter-assay CV: 15%; detection limit: 0.10 pg/ml).

Outcomes

Cardiovascular Disease (CVD)

The outcome was self-reported CVD of atherosclerotic origin. Self-reported CVD was based on the reported presence of heart diseases, stroke, and surgical interventions. Diseases

included coronary heart disease, angina pectoris, heart failure, cardiac arrhythmia, other heart diseases. Stroke was identified through the question, "Did you ever have a stroke?", with confirmation based on the use of medication for this condition. Surgical interventions inquired about included heart or coronary vessel surgeries, specifically asking, "Did you have an operation on your heart or coronary arteries?" with further prompts for details such as heart valve replacement, coronary bypass, balloon treatment, pacemaker insertion, or other types of cardiac surgery. Only participants who reported one or more of these diseases, or surgical interventions and were concurrently using cardiovascular medications were categorized as having CVD.

UK Biobank (UKB)

Outcomes

CIDI-MDD (data field 20446, 20441, 20449, 20536, 20532, 20435, 20450, 20437), and PHQ-9 (data field 20514, 20507, 20510, 20508, 20517, 20518, 20519, 20511, 20513).

Covariates

Further information is available online from relevant UKB data fields per covariate; sex (data field 31), age (data field 21003), Townsend Deprivation Index (data field 189), smoking (data field 20116), frequency of drinking alcohol (data field 20414), physical activity (data field 884), and BMI (data field 21001).

Supplement Methods

Outlier detection

We applied the Isolation Forest algorithm (2) to detect outliers. The algorithm works by isolating anomalies instead of profiling normal data points; it randomly selects a feature and splits the data between the maximum and minimum values, repeating this process recursively.

Machine Learning framework

To address the class imbalance in the machine learning model, we used Synthetic Minority Over-sampling Technique (SMOTE) to oversample the minority class (3). We fine-tuned the model's hyperparameters using Optuna (4), aiming to maximize the weighted F1 score through 5-fold stratified cross-validation, which helped reduce variance in performance estimates.

To determine feature importance, we calculated the original importance scores for each attribute using Extreme Gradient Boosting’s (XGBoost) built-in feature importance metric, which quantifies each feature's contribution to model accuracy by assessing how frequently it is used in node splits across all trees, weighted by the reduction in impurity these splits achieve. To assess the stability of these importance scores, we employed bootstrapping with replacement (1000 iterations). In each iteration, a random sample of the dataset was drawn with replacement, and the XGBoost model was re-trained on this resampled data. This process allowed us to calculate a distribution of importance scores and standard deviation for each attribute.

For statistical significance testing of each feature, we used a permutation test. This involved randomly shuffling the values of each feature across samples and re-evaluating the model’s performance, creating a distribution of importance values under the null hypothesis. We calculated p-values for each feature to assess the likelihood that their observed importance was due to chance, using a significance threshold of 0.05.

Causal Discovery

Constraint-based causal discovery algorithms are a class of methods used to infer causal relationships between variables from observational data. These algorithms rely on conditional independence tests to identify causal structures that are consistent with the observed statistical dependencies in the data. One of the most well-known constraint-based

algorithms is the PC (Peter-Clark) algorithm. The PC algorithm works as follows: start with a fully connected undirected graph; use conditional independence tests to remove edges between variables that are conditionally independent; orient edges to form a Causal Graph (CG) based on the remaining dependencies. Constraint-based algorithms aim to find CGs that are consistent with the observed conditional independence relations in the data. They typically assume causal Markov condition (5), the causal sufficiency (no unmeasured confounders) and faithfulness (6).

Supplement Results

Machine Learning results

The machine learning model achieved an overall accuracy 74%, with a macro and weighted average (precision, recall, and F1-score) 0.74. Additionally, the model's ROC curve had an area under the curve (AUC) 0.81, indicating high power in distinguishing between MDD and non-MDD cases (Supplementary Figure S2). The model correctly classified 130 true positives (MDD cases) and 134 true negatives (No MDD cases), with 51 false positives and 40 false negatives.

Causal Graph Analysis, Stability results

Stability analysis highlighted strong connections in pathways from current MDD (cause) to key biomarkers (effect). Most biomarkers showed stability across all three alpha thresholds examined, with two exceptions: LDL triglycerides were stable only at medium alpha levels (0.05 and 0.075), while IL-6 demonstrated stability solely at the highest alpha tested (0.075). In pathways leading from biomarkers to CVD (effect), the 10 biomarkers appearing in 82.3% to 99.7% of the BGs (median 97.0%). The evaluation of alpha conditions for each biomarker leading to CVD indicated consistent presence across these thresholds, with all biomarkers appearing at all three alpha levels, except for HDL diameter and LDL triglycerides, which was absent at every tested alpha level.

Supplement Tables/Figures

Table S1. Biomarkers and their groupings by measurement method.

Category	Biomarker (unit)	Measurement Method	Notes
**NMR-Measured Metabolites**			
Amino acids	Alanine (mmol/L)	NMR spectroscopy	-
	Glutamine (mmol/L)	NMR spectroscopy	-
	Histidine (mmol/L)	NMR spectroscopy	-
	Isoleucine (mmol/L)	NMR spectroscopy	-
	Leucine (mmol/L)	NMR spectroscopy	-
	Phenylalanine (mmol/L)	NMR spectroscopy	-
	Tyrosine (mmol/L)	NMR spectroscopy	-
	Valine (mmol/L)	NMR spectroscopy	-
Apolipoproteins	Apolipoprotein A-I (g/L)	NMR spectroscopy	-
	Apolipoprotein B (g/L)	NMR spectroscopy	-
Cholesterol	Esterified cholesterol (mmol/L)	NMR spectroscopy	-
	Free cholesterol (mmol/L)	NMR spectroscopy	-
	HDL cholesterol (mmol/L)	NMR spectroscopy	-
	HDL2 cholesterol (mmol/L)	NMR spectroscopy	-
	HDL3 cholesterol (mmol/L)	NMR spectroscopy	-
	LDL cholesterol (mmol/L)	NMR spectroscopy	-
	Remnant cholesterol (mmol/L)	NMR spectroscopy	-
	Serum total cholesterol (mmol/L)	NMR spectroscopy	-
	VLDL cholesterol (mmol/L)	NMR spectroscopy	-
Fatty acids	Conjugated linoleic acids (mmol/L)	NMR spectroscopy	-
	Docosahexaenoic acid, 22:6 (mmol/L)	NMR spectroscopy	-
	Linoleic acid, 18:2 (mmol/L)	NMR spectroscopy	-
	Monounsaturated fatty acids (mmol/L)	NMR spectroscopy	-
	Omega-3 fatty acids (mmol/L)	NMR spectroscopy	-
	Omega-6 fatty acids (mmol/L)	NMR spectroscopy	-
	Polyunsaturated fatty acids (mmol/L)	NMR spectroscopy	-
	Saturated fatty acids (mmol/L)	NMR spectroscopy	-
Fluid balance	Albumin (signal area)	NMR spectroscopy	-
	Creatinine (mmol/L)	NMR spectroscopy	-
Glycerides & phospholipids	Phosphatidylcholine and other cholines (mmol/L)	NMR spectroscopy	-
	Serum total triglycerides (mmol/L)	NMR spectroscopy	-
	Sphingomyelins (mmol/L)	NMR spectroscopy	-
	Total cholines (mmol/L)	NMR spectroscopy	-
	Total phosphoglycerides (mmol/L)	NMR spectroscopy	-
	Triglycerides in HDL (mmol/L)	NMR spectroscopy	-
	Triglycerides in LDL (mmol/L)	NMR spectroscopy	-
	Triglycerides in VLDL (mmol/L)	NMR spectroscopy	-
Glycolysis-related	Citrate (mmol/L)	NMR spectroscopy	-
	Glucose (mmol/L)	NMR spectroscopy	-
	Lactate (mmol/L)	NMR spectroscopy	-
Inflammation (NMR)	Glycoprotein acetyls (mmol/L)	NMR spectroscopy	-
Ketone bodies	3-hydroxybutyrate (mmol/L)	NMR spectroscopy	-
	Acetate (mmol/L)	NMR spectroscopy	-
	Acetoacetate (mmol/L)	NMR spectroscopy	-
Lipoprotein particle size	Mean diameter for HDL particles (nm)	NMR spectroscopy	-

	Mean diameter for LDL particles (nm)	NMR spectroscopy	-
	Mean diameter for VLDL particles (nm)	NMR spectroscopy	-
Fatty acid saturation	Estimated degree of unsaturation	NMR spectroscopy	-
	Estimated fatty acid chain length	NMR spectroscopy	-
	Total fatty acids (mmol/L)	NMR spectroscopy	-
**ELISA-Measured Inflammatory Markers**			
Inflammation	CRP (mg/L)	ELISA (Dako antibodies)	Clinical Chemistry department of the VU University Medical Center
	IL-6 (pg/mL)	ELISA (Sanquin PeliKine)	Clinical Chemistry department of the VU University Medical Center
	TNF-α (pg/mL)	ELISA (R&D Systems Quantikine)	Processed at Good Biomarker Science, (Leiden, The Netherlands)

Table S2. Frequency distribution of PHQ-9 symptom severity and missingness in UK Biobank participants.

**Characteristics**	**UK Biobank, *N = 157,286***				
PHQ-9					
	**Not at all, *%***	**Several days, *%***	**More than half the days, *%***	**Nearly every day, *%***	**Missing, %**
Anhedonia	80.94	14.93	2.24	1.62	0.26
Depressed mood	77.35	18.87	2.04	1.39	0.34
Sleep problems	50.78	34.08	7.07	7.87	0.2
Fatigue	50.03	38.96	5.46	5.35	0.21
Appetite changes	81.46	13.15	2.8	2.45	0.13
Feelings of inadequacy	79.87	15.38	2.2	2.07	0.47
Cognitive problems	81.76	14.44	2.04	1.62	0.15
Psychomotor changes	94.32	4.17	0.78	0.58	0.16
Suicidal ideation	95.08	3.39	0.43	0.34	0.74

Table S3. UK Biobank ICD-10 codes for cardiovascular disease endpoints included in analysis: code definitions and categories

ICD-10	Meaning	Category
G463	Brain stem stroke syndrome	G46 Vascular syndromes of brain in cerebrovascular diseases
G464	Cerebellar stroke syndrome	G46 Vascular syndromes of brain in cerebrovascular diseases
I200	Unstable angina	I20 Angina pectoris
I201	Angina pectoris with documented spasm	I20 Angina pectoris
I208	Other forms of angina pectoris	I20 Angina pectoris
I209	Angina pectoris, unspecified	I20 Angina pectoris
I210	Acute transmural myocardial infarction of anterior wall	I21 Acute myocardial infarction
I211	Acute transmural myocardial infarction of inferior wall	I21 Acute myocardial infarction
I212	Acute transmural myocardial infarction of other sites	I21 Acute myocardial infarction
I213	Acute transmural myocardial infarction of unspecified site	I21 Acute myocardial infarction
I214	Acute subendocardial myocardial infarction	I21 Acute myocardial infarction
I219	Acute myocardial infarction, unspecified	I21 Acute myocardial infarction
I220	Subsequent myocardial infarction of anterior wall	I22 Subsequent myocardial infarction
I221	Subsequent myocardial infarction of inferior wall	I22 Subsequent myocardial infarction
I228	Subsequent myocardial infarction of other sites	I22 Subsequent myocardial infarction
I229	Subsequent myocardial infarction of unspecified site	I22 Subsequent myocardial infarction
I230	Haemopericardium as current complication following acute myocardial infarction	I23 Certain current complications following acute myocardial infarction
I231	Atrial septal defect as current complication following acute myocardial infarction	I23 Certain current complications following acute myocardial infarction
I232	Ventricular septal defect as current complication following acute myocardial infarction	I23 Certain current complications following acute myocardial infarction
I233	Rupture of cardiac wall without haemopericardium as current complication following acute myocardial infarction	I23 Certain current complications following acute myocardial infarction
I234	Rupture of chordae tendineae as current complication following acute myocardial infarction	I23 Certain current complications following acute myocardial infarction
I235	Rupture of papillary muscle as current complication following acute myocardial infarction	I23 Certain current complications following acute myocardial infarction
I236	Thrombosis of atrium, auricular appendage, and ventricle as current complications following acute myocardial infarction	I23 Certain current complications following acute myocardial infarction
I238	Other current complications following acute myocardial infarction	I23 Certain current complications following acute myocardial infarction
I240	Coronary thrombosis not resulting in myocardial infarction	I24 Other acute ischaemic heart diseases
I248	Other forms of acute ischaemic heart disease	I24 Other acute ischaemic heart diseases
I249	Acute ischaemic heart disease, unspecified	I24 Other acute ischaemic heart diseases
I252	Old myocardial infarction	I25 Chronic ischaemic heart disease
I255	Ischaemic cardiomyopathy	I25 Chronic ischaemic heart disease
I258	Other forms of chronic ischaemic heart disease	I25 Chronic ischaemic heart disease
I259	Chronic ischaemic heart disease, unspecified	I25 Chronic ischaemic heart disease
I630	Cerebral infarction due to thrombosis of precerebral arteries	I63 Cerebral infarction

I632	Cerebral infarction due to unspecified occlusion or stenosis of precerebral arteries	I63 Cerebral infarction
I633	Cerebral infarction due to thrombosis of cerebral arteries	I63 Cerebral infarction
I635	Cerebral infarction due to unspecified occlusion or stenosis of cerebral arteries	I63 Cerebral infarction
I700	Atherosclerosis of aorta	I70 Atherosclerosis
I7000	Atherosclerosis of aorta (without gangrene)	I70 Atherosclerosis
I7001	Atherosclerosis of aorta (with gangrene)	I70 Atherosclerosis
I701	Atherosclerosis of renal artery	I70 Atherosclerosis
I7010	Atherosclerosis of renal artery (without gangrene)	I70 Atherosclerosis
I7011	Atherosclerosis of renal artery (with gangrene)	I70 Atherosclerosis
I702	Atherosclerosis of arteries of the extremities	I70 Atherosclerosis
I7020	Atherosclerosis of arteries of extremities (without gangrene)	I70 Atherosclerosis
I7021	Atherosclerosis of arteries of extremities (with gangrene)	I70 Atherosclerosis
I708	Atherosclerosis of other arteries	I70 Atherosclerosis
I7080	Atherosclerosis of other arteries (without gangrene)	I70 Atherosclerosis
I7081	Atherosclerosis of other arteries (with gangrene)	I70 Atherosclerosis
I709	Generalised and unspecified atherosclerosis	I70 Atherosclerosis
I7090	Generalized and unspecified atherosclerosis (without gangrene)	I70 Atherosclerosis
I7091	Generalized and unspecified atherosclerosis (with gangrene)	I70 Atherosclerosis
Z951	Presence of aortocoronary bypass graft	Z95 Presence of cardiac and vascular implants and grafts
Z955	Presence of coronary angioplasty implant and graft	Z95 Presence of cardiac and vascular implants and grafts

Table S4. Evaluation metrics of the Extreme Gradient Boosting (XGBoost) model for classifying current Major Depressive Disorder (MDD).

Metric	Class 0 (No MDD)	Class 1 (MDD)	Overall
Precision	0.79	0.72	-
Recall	0.72	0.77	-
F1-Score	0.74	0.75	-
Accuracy	-	-	0.74
Sensitivity	-	-	0.77
Specificity	-	-	0.72
Macro Average (Precision/Recall/F1)	-	-	0.74
Weighted Average (Precision/Recall/F1)	-	-	0.74
Receiver Operating Characteristic (ROC) curve			0.81
True Positives (TP)	-	130	-
True Negatives (TN)	134	-	-
False Positives (FP)	51	-	-
False Negatives (FN)	-	40	-

Table S5. Descriptive statistics and missingness of the filtered biomarkers in NESDA.

Biomarkers	*Baseline*	
	**N=2,981**	**Missing, %**
Inflammatory markers (mean ± SD)		
**C-Reactive Protein (mg/L)**	2.82 (5.06)	1.5
**Interleukin 6 (pg/L)**	1.54 (13.38)	1.47
**Tumor Necrosis Factor-alpha (pg/L)**	1.09 (1.4)	2.11
**Glycoprotein acetyls** *(mmol/l)*	1.34 (0.2)	2.25
Apolipoproteins (mean ± SD)		
**Apolipoprotein A1** *(g/l)*	1.62 (0.19)	2.25
**Apolipoprotein B** *(g/l)*	0.96 (0.23)	2.25
Cholesterol (mean ± SD)		
**Remnant cholesterol** *(mmol/l)*	1.61 (0.45)	2.25
**VLDL cholesterol** *(mmol/l)*	0.86 (0.29)	2.25
**HDL cholesterol** *(mmol/l)*	1.4 (0.33)	2.25
**HDL2 cholesterol** *(mmol/l)*	0.92 (0.3)	2.25
**HDL3 cholesterol** *(mmol/l)*	0.48 (0.05)	2.25
**Mean diameter of VLDL** *(nm)*	36.43 (1.28)	2.25
**Mean diameter of HDL** *(nm)*	10.02 (0.24)	2.25
Diglycerides and Triglycerides (mean ± SD)		
**Diglycerides** *(mmol/l)*	0.02 (0.02)	4.02
**Serum total TG** *(mmol/l)*	1.23 (0.66)	2.25
**VLDL TG** *(mmol/l)*	0.86 (0.58)	2.25
**LDL TG** *(mmol/l)*	0.14 (0.06)	2.25
**HDL TG** *(mmol/l)*	0.13 (0.04)	2.25
Fatty Acids (FA) (mean ± SD)		
**Monounsaturated FA** *(mmol/l)*	2.83 (0.94)	2.35
**Total FA** *(mmol/l)*	11.47 (2.53)	2.31
**Estimated FA chain length**	17.41 (0.36)	2.31
Ketone Bodies (mean ± SD)		
**Acetate** *(mmol/l)*	0.05 (0.05)	2.3

Amino Acids (mean ± SD)		
**Tyrosine** (mmol/l)	0.06 (0.01)	2.25
**Isoleucine** *(mmol/l)*	0.05 (0.02)	2.5

Table S6. Multidisciplinary research team composition and their affiliations.

Jos A. Bosch	• Department of Clinical Psychology, University of Amsterdam, Amsterdam, The Netherlands.<br>• Department of medical Psychology, Amsterdam University Medical Centers, Amsterdam, The Netherlands.
Femke Lamers	• Department of Psychiatry, Amsterdam UMC location Vrije Universiteit Amsterdam, Boelelaan 1117, Amsterdam, The Netherlands.<br>• Amsterdam Public Health, Mental Health Program, Amsterdam The Netherlands.
Yuri Milaneschi	• Department of Psychiatry, Amsterdam UMC location Vrije Universiteit Amsterdam, Boelelaan 1117, Amsterdam, The Netherlands.<br>• Amsterdam Public Health, Mental Health Program, Amsterdam The Netherlands.<br>• Amsterdam Public Health, Methodology Program, Amsterdam, The Netherlands.<br>• Amsterdam Neuroscience, Mood, Anxiety, Psychosis, Sleep & Stress program, Amsterdam, The Netherlands.<br>• Amsterdam Neuroscience, Complex Trait Genetics, Amsterdam, The Netherlands.
Karina Nowakowska	• Department of Old Age Psychiatry and Psychotic Disorders, Medical University of Lodz (Poland).
Stefanos Bellos	• Department of Psychiatry, Ioannina Medical School, Greece.
Panagiotis Simos	• Professor, Developmental Neuropsychology, Department of Psychiatry & Behavioral Science, Medical School, University of Crete, Greece.
Jakub Kazmierski	• Department of Old Age Psychiatry and Psychotic Disorders, Medical University of Lodz (Poland).
Winfried März	• Department of Internal Medicine V, University of Heidelberg, Mannheim, Germany.<br>• Clinical Institute of Medical and Chemical Laboratory Diagnostics, Medical University of Graz, Austria.<br>• SYNLAB Holding Deutschland GmbH, Augsburg, Germany.
Aikaterini Nakka	• Department of Cardiology, Faculty of Medicine, School of Health Sciences, University of Ioannina, Greece.
Sander W. van der Laan	• Central Diagnostic Laboratory, Division Laboratories, Pharmacy, and Biomedical genetics, University Medical Center Utrecht, Utrecht University, the Netherlands.<br>• Department of Genomic Sciences, University of Virginia, Charlottesville, VA, USA.

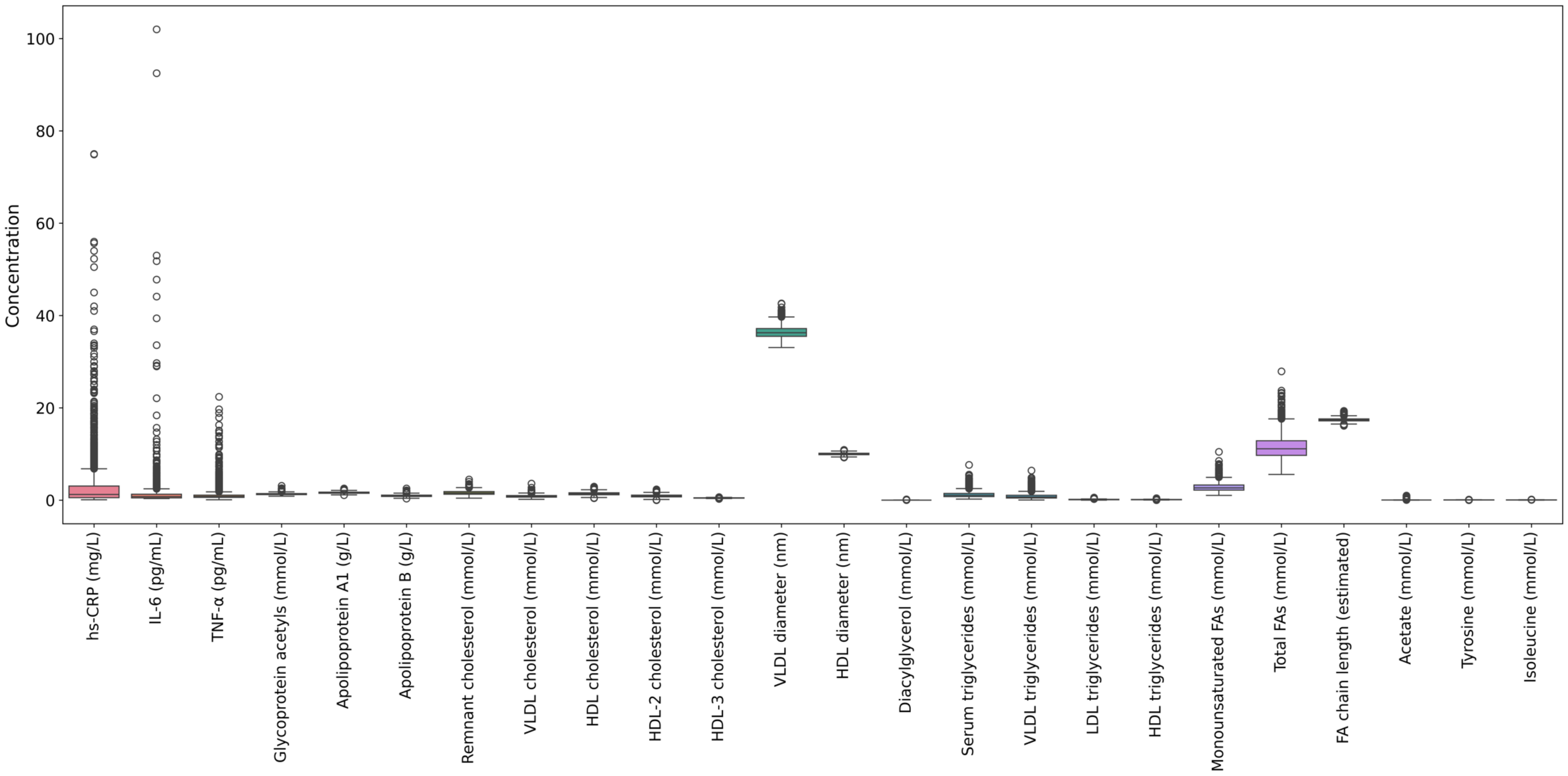

Figure S1 Box plot visualization of biomarker values after applying the Isolation Forest algorithm to detect and remove outliers. Each box plot represents the spread of values of a specific biomarker, with outliers shown as individual points.

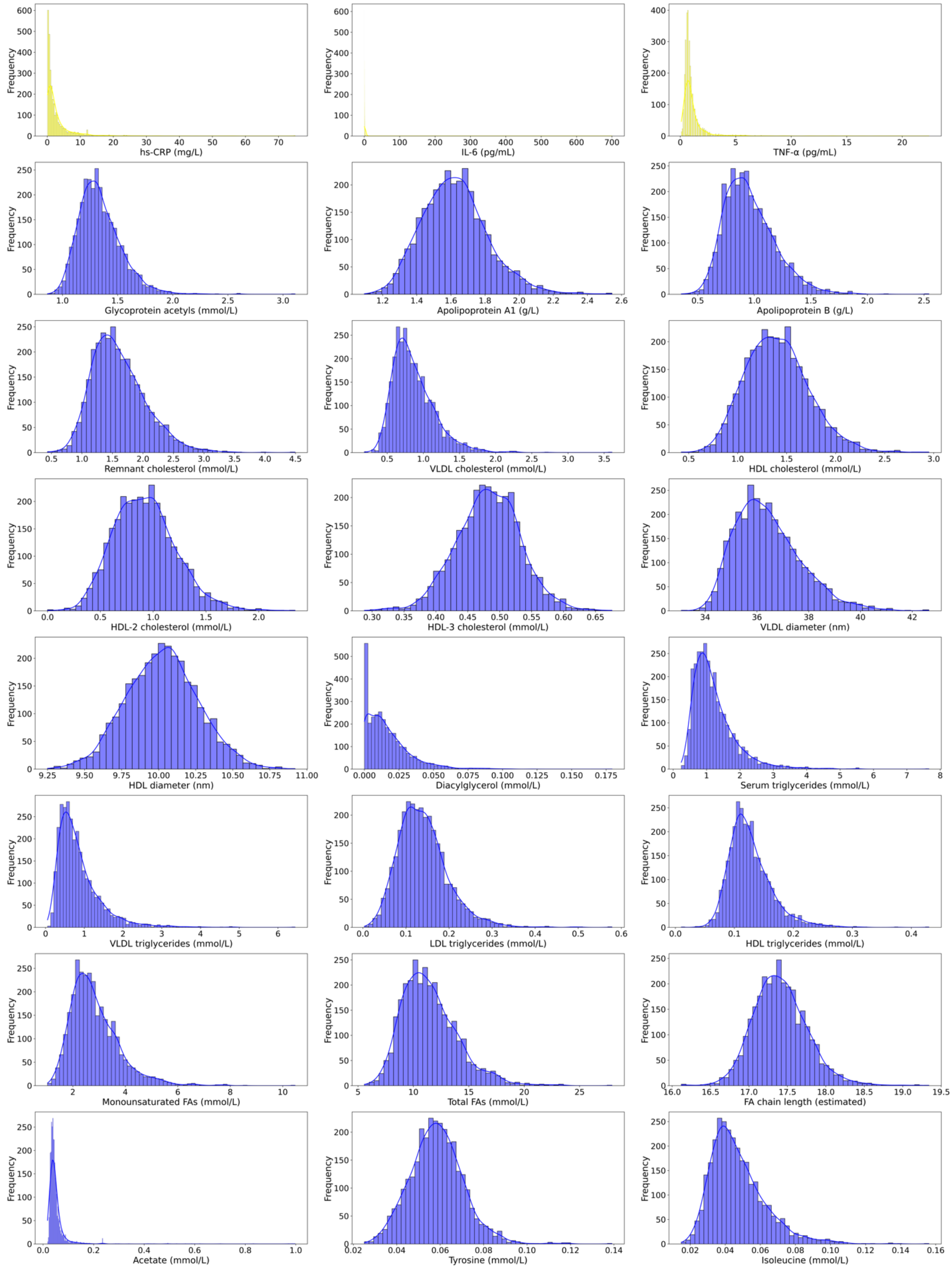

Figure S2 Distribution plots of refined metabolites and inflammatory markers.

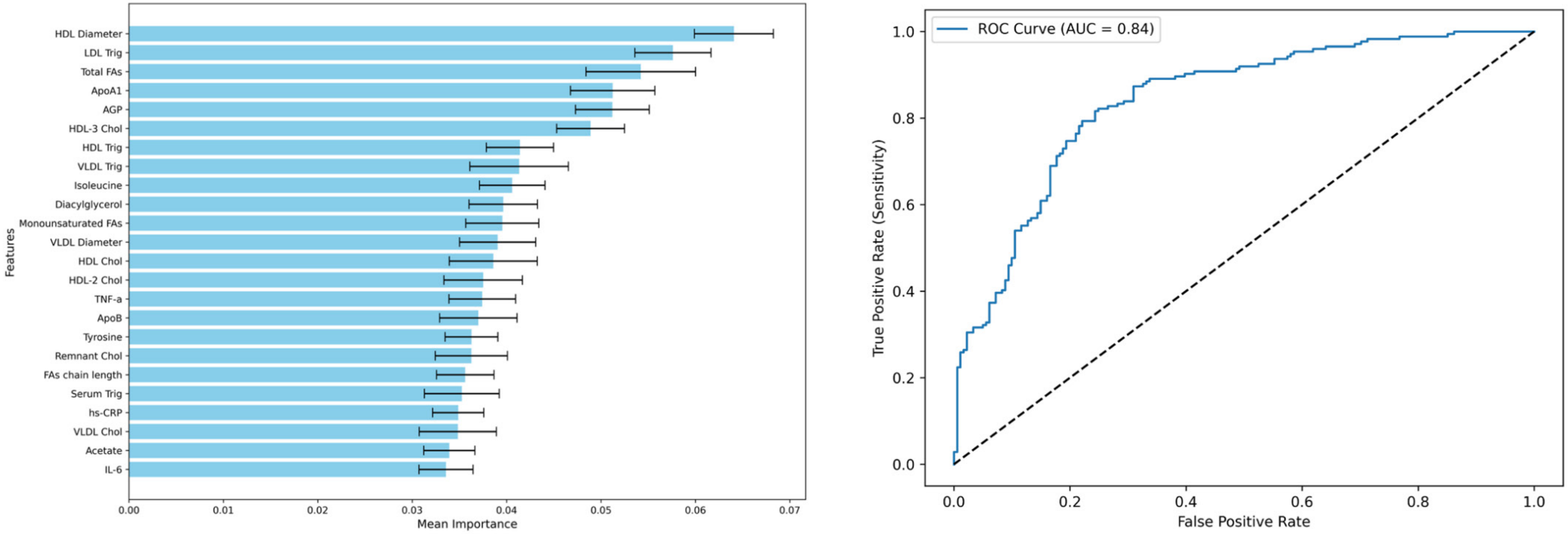

A.

B.

Figure S3. A. Sorted feature importance of biomarkers used in the prediction model for current Major Depressive Disorder (MDD) classification after sensitivity analysis. The bars represent the mean feature importance scores, with error bars indicating the standard deviation of each marker's importance. B. Receiver Operating Characteristic (ROC) curve for the prediction of MDD using the Extreme Gradient Boosting (XGBoost) model. The curve illustrates the model's performance, with an area under the curve (AUC) of 0.81, indicating strong discrimination between MDD and non-MDD cases. The orange line represents the ROC curve, and the dashed blue line represents the line of no-discrimination (AUC = 0.5), which serves as a reference.

References

1. Penninx BWJH, Beekman ATF, Smit JH, Zitman FG, Nolen WA, Spinhoven P, *et al.* (2008): The Netherlands Study of Depression and Anxiety (NESDA): rationale, objectives and methods. *Int J Methods Psychiatr Res* 17: 121–140.
2. Cortes D (2021, November 23): Isolation forests: looking beyond tree depth [no. arXiv:2111.11639]. arXiv. https://doi.org/10.48550/arXiv.2111.11639
3. Chawla NV, Bowyer KW, Hall LO, Kegelmeyer WP (2002): SMOTE: Synthetic Minority Over-sampling Technique. *J Artif Intell Res* 16: 321–357.
4. Akiba T, Sano S, Yanase T, Ohta T, Koyama M (2019, July 25): Optuna: A Next-generation Hyperparameter Optimization Framework [no. arXiv:1907.10902]. arXiv. https://doi.org/10.48550/arXiv.1907.10902
5. Janzing D, Scholkopf B (2010): Causal Inference Using the Algorithmic Markov Condition. *IEEE Trans Inf Theory* 56: 5168–5194.
6. Glymour C, Zhang K, Spirtes P (2019): Review of Causal Discovery Methods Based on Graphical Models. *Front Genet* 10: 524.
